# Building an Efficient Peritoneal Surface Malignancies Program Despite the Lower-Middle–Income Barriers: Ukraine Experience

**DOI:** 10.1200/GO.23.00432

**Published:** 2024-02-08

**Authors:** Viacheslav Kopetskyi, Marta Antoniv, Roman Yarema, Viacheslav Maksymovskyi, Valeriia Chetverikova-Ovchinnik, Vitalii Kryzhevskyi, Nataliya Volodko, Vadim Gushchin, Andrei Nikiforchin

**Affiliations:** ^1^ Department of Hepatopancreatobiliary Surgery, National Cancer Institute, Kyiv, Ukraine; ^2^ Department of Surgery, Ordensklinikum Linz, Linz, Austria; ^3^ Department of Oncology, Danylo Halytsky Lviv National Medical University, Lviv, Ukraine; ^4^ Department of Surgery 1, Odesa National Medical University, Odesa, Ukraine; ^5^ Department of General and Military Surgery, Odesa National Medical University, Odesa, Ukraine; ^6^ Department of Surgical Oncology, Mercy Medical Center, Baltimore, MD

## Abstract

**PURPOSE:**

Cytoreductive surgery/hyperthermic intraperitoneal chemotherapy (CRS/HIPEC) programs are often limited to centers in developed countries because of extensive requirements. We aimed to analyze efficacy and challenges of CRS/HIPEC centers in lower-middle–income settings in the Ukraine example.

**METHODS:**

A multicenter descriptive study was conducted using data sets (2008-2022) from Kyiv, Lviv, and Odesa centers. Patients with appendiceal neoplasm (AN); colorectal cancer (CRC); malignant peritoneal mesothelioma (MPM); and epithelial ovarian, fallopian tube, and primary peritoneal cancer (EOC) treated with CRS ± HIPEC were included. Overall survival (OS) was analyzed for N ≥ 20 cohorts using the Kaplan-Meier method.

**RESULTS:**

We included 596 patients. At Kyiv and Lviv centers, 37 and 28 patients with AN had completeness of cytoreduction (CC-0/1) rates of 84% and 71%, respectively. Thirty-day major morbidity stood at 24% and 18%, respectively. Median OS was not reached (NR) at both centers. Nineteen patients with CRC from Kyiv, 11 from Lviv, and 156 from Odesa had CC‐0/1 rates of 84%, 91%, and 86%, respectively. Thirty-day major complications occurred in 16%, 18%, and 8%, respectively. Median OS in the Odesa cohort was 35 (95% CI, 32 to 38) months. Among 15 Kyiv, five Lviv, and six Odesa patients with MPM, CC‐0/1 rates were 67%, 80%, and 100%, respectively, while major complications occurred in 13%, 0%, and 17%, respectively. OS was not analyzed because of small MPM cohorts. At Kyiv, Lviv, and Odesa centers, 91, 40, and 89 patients, respectively, had primary EOC. CC-0/1 rates were 79%, 100%, and 80%, and 30-day major morbidity rates were 23%, 5%, and 6%, respectively. Median OS was NR, 71 (95% CI, 32 to 110), and 67 (95% CI, 61 to 73) months, respectively.

**CONCLUSION:**

CRS/HIPEC programs in lower-middle–income environment can achieve safety and survival that meet global standards. Our discussion highlights common obstacles in such settings and proposes effective overcoming strategies.

## Teaser

Explore overcoming #LMIC barriers & achieving standards in #PSMmanagement on example! #HIPEC

## INTRODUCTION

Establishing and maintaining a cytoreductive surgery/hyperthermic intraperitoneal chemotherapy (CRS/HIPEC) program is a demanding undertaking. It requires not only skilled surgical, anesthesia, and intensive care unit (ICU) teams but also meticulous patient selection, comprehensive postoperative care, regular follow-up, and ongoing research.^1-3^ Under these circumstances, CRS/HIPEC becomes a potentially curative treatment for various peritoneal surface malignancies (PSMs) including appendiceal, colorectal, ovarian, and primary peritoneal neoplasms.^4-8^ Because of the described extensive requirements, CRS/HIPEC is typically performed in specialized centers with their wealth of experience and resources leading to superior short- and long-term outcomes.^8-10^ As a result, PSM programs are predominantly found in developed countries and may not always be replicated in other settings.

**CONTEXT**

**Key Objective**
Is it feasible to establish an efficient peritoneal surface malignancy (PSM) program in lower-middle–income countries, given their socioeconomic and infrastructural challenges?
**Knowledge Generated**
The Ukrainian example demonstrates that PSM programs launched in economically constrained settings can attain safety and survival outcomes matching global standards. This was accomplished through targeted strategies including codeveloping national guidelines by medical community and health care officials, enhancing PSM awareness among physicians and the public, and promoting multidisciplinary collaboration for referral and follow-up.
**Relevance**
This study offers a practical blueprint for establishing effectively functioning PSM programs in comparable socio-economic contexts, thus expanding the availability of advanced oncological care to patients.

In lower-middle–income countries (LMICs), starting a CRS/HIPEC program presents specific challenges dictated by socioeconomic factors.^11,12^ They include suboptimal medical training, insufficient health care expenditure, governmental policies, and inadequate patient follow-up strategies, among others.^13^ The historical background, especially in post-Soviet countries, also contributes to the equation. Persistent issues such as the quality of medical education, a prevailing paternalistic decision-making model, limited self-conducted research, and disorganized patient logistics only exacerbate existing economic complexities.^9,14^ Thus, the development of a robust CRS/HIPEC program is hindered, potentially denying patients with PSM access to appropriate, high-quality care.

We chose to address the issue of overcoming existing barriers by focusing on the specific case of Ukraine.^15^ Our aim was to analyze the Ukrainian experience and outline certain strategies that could facilitate the development of CRS/HIPEC centers in regions facing similar hurdles.

## METHODS

### Study Design, Settings, and Data Source

We designed and conducted a multicenter descriptive study to assess the process of building and maintaining CRS/HIPEC programs in LMICs and analyze their outcomes. There are three operating PSM centers in Ukraine—Kyiv, Lviv, and Odesa. Table 1 provides their detailed description according to the Chicago Consensus Standards.^16^ We reviewed the centers' prospectively maintained data sets (2008-2022) and included patients with peritoneal spread of appendiceal neoplasms (ANs); colorectal cancer (CRC); malignant peritoneal mesothelioma (MPM); and epithelial ovarian, fallopian tube, and primary peritoneal cancer (EOC), who underwent a CRS with or without HIPEC. The Lviv data set had only patients treated with CRS/HIPEC. Patients with lacked data, aborted surgeries, gastric cancer, endometrial carcinoma, neuroendocrine tumors, and sarcoma were excluded from the analysis.^17^ Before any procedure, all patients signed an informed consent for the use of their nonidentifiable data approved by the centers' institutional review boards.

**TABLE 1:** PSM Centers Characteristics

Variable	Kyiv	Lviv	Odesa
Starting year of the PSM program	2017	2008	2013
Source of the program funding	Government Out-of-pocket payments	Government Out-of-pocket payments	Government
Institution type	Academic	Academic	Academic
Tumor board	Yes	Yes	Yes
Pathologists with training in PSM	No	Yes	Yes
Radiologists with training in PSM	No	Yes	Yes
Specialists in nutrition, OT, and PT	No	No	No
24-hour access to			
Surgical ICU	Yes	Yes	Yes
Blood bank	Yes	Yes	Yes
Computed tomography	Yes	No (radiologist can be summoned)	Yes
Interventional radiology	Yes	No	Yes
Endoscopy	Yes	No	Yes
Institutional review board	Yes	Yes	Yes
PSM research department or team	No	No	No
Maintained PSM database	Yes	Yes	Yes
Participation in RCTs	No	No	No

Abbreviations: ICU, intensive care unit; OT, occupational therapy; PSM, peritoneal surface malignancies; PT, physical therapy; RCT, randomized controlled trial.

### Scores and Scales

Tumor burden was evaluated intraoperatively using the peritoneal cancer index (PCI), with scores ranging from 0 to 39, with PCI scores ≥20 considered extensive disease.^18^ Completeness of cytoreduction (CC) was assessed with a CC score, where CC-0 stands for no visible residual tumor, CC-1—residual lesions <2.5 mm, CC-2—residual lesions from 2.5 mm to 2.5 cm, and CC-3—residual lesions >2.5 cm.^19^ We considered cytoreductions with CC-0/1 complete. Postoperative 30-day morbidity and 60-day mortality were evaluated using the Clavien-Dindo classification with grades 3-4 considered major complications.^20^

### Diagnosis and CRS/HIPEC

Before CRS/HIPEC, the diagnosis was pathologically confirmed and tumor markers were assessed in most patients. Chest, abdominal, and pelvic computed tomography (CT) was performed in everyone. A routine diagnostic laparoscopy preceding CRS was used in all patients at the Odesa center, in patients with MPM and EOC at the Lviv center, and was not used in Kyiv.

Anesthesia used intravenous and inhalation agents along with neuromuscular blocking drugs; epidural anesthesia and temperature monitoring were used routinely at all centers. CRS started with a midline incision and PCI recording. Peritonectomies and organ resections were performed as needed to achieve CC. Upon the end of the surgery, CC score was recorded and in HIPEC patients, chemoperfusion was done with a closed technique (regimens are in Table 2), while all anastomoses were performed afterward. An operative note was filled out within 24 hours after CRS/HIPEC and the pathology report was ready within 2-4 weeks. Additional procedure details occurred as previously described.^21,22^

**TABLE 2:** Surgeons and Procedures

Variable	Kyiv	Lviv	Odesa
No. of PSM surgeons	2	3	5
Years of experience	9 and 20 years	15, 20, and 30 years	From 15 to 30 years
PSM surgeons training	General surgery Surgical oncology Gynecology	General surgery Surgical oncology	General surgery Surgical oncology
International training in PSM	Mercy Medical Center (Baltimore, MD) North Hampshire Hospital (Basingstoke, United Kingdom) Sheba Medical Center (Ramat Gan, Israel)	Lyon Sud Hospital Center (Lyon, France) North Hampshire Hospital (Basingstoke, United Kingdom) ESSO CRS/HIPEC Course (Hamburg, Germany)	National Cancer Institute (Milan, Italy)
No. of cases per surgeon a year	5-15 and 40-50 cases	10-50 cases	8-43 cases
Assistants in the OR	Another surgeon Surgical resident	Another surgeon	Another surgeon Surgical resident
HIPEC agent is prepared by	PSM surgeon	PSM surgeon	PSM surgeon
HIPEC agents and regimens (administered once at chemoperfusion)	Mitomycin C (30 mg/m^2^); temp 42°C—90 minutes Mitomycin C (20 mg/m^2^) + cisplatin (25 mg/m^2^); temp 42°C-43°C—90 minutes Doxorubicin (15 mg/m^2^) + cisplatin (50 mg/m^2^); temp 42°C—90 minutes Cisplatin (100 mg/m^2^); temp 42°C—60 minutes	Mitomycin C (30 mg/m^2^); temp 42°C-43°C—90 minutes Mitomycin C (20 mg/m^2^) + cisplatin (25 mg/m^2^); temp 42°C-43°C—90 minutes Doxorubicin (15 mg/m^2^) + cisplatin (75 mg/m^2^); temp 42°C-43°C—90 minutes Cisplatin (100 mg/m^2^); temp 42°C—90 minutes	Mitomycin C (30 mg/m^2^); temp 42°C—90 minutes Doxorubicin (15 mg/m^2^) + cisplatin (50 mg/m^2^); temp 42°C—90 minutes Carboplatin (800 mg/m^2^); temp 42°C—90 minutes
HIPEC is performed and monitored by	PSM surgeon Surgical resident	PSM surgeon Anesthesiologist	PSM surgeon

Abbreviations: CRS, cytoreductive surgery; ESSO, European Society of Surgical Oncology; HIPEC, hyperthermic intraperitoneal chemotherapy; OR, operating room; PSM, peritoneal surface malignancies; temp, temperature.

### Statistical Analysis

Statistical analysis was conducted using the IBM SPSS Statistics software (version 23.0; IBM Corporation; Armonk, NY; for identification only). Survival analysis was performed for cohorts with ≥20 patients using the Kaplan-Meier method. Overall survival (OS) was defined as the time from surgery to the date of death from any cause. Progression-free survival (PFS) was assessed only in CC-0/1 patients and defined as the time from the procedure to disease recurrence or death, whichever occurred first. Statistical significance was defined as *P* < .05.

## RESULTS

We included 596 patients: 172 from the Kyiv center, 171 from the Lviv center, and 253 from the Odesa center.

### ANs

Overall, 37 patients with AN from the Kyiv center and 28 from the Lviv center were included (Table 3). The characteristics of one patient with AN from the Odesa center are provided in Table 3 and not described here. The median PCI score was 21 (IQR, 13-30) at the Kyiv center and 33 (IQR, 18-36) at the Lviv center. The CC‐0/1 rate was 84% in Kyiv patients and 71% in Lviv patients. Thirty-day major complications occurred in 24% (n = 9) and 18% (n = 5), respectively.

**TABLE 3:** Perioperative Characteristics of Patients With AN

Variable	PSM Center		
	Kyiv (n = 37)	Lviv (n = 28)	Odesa (n = 1)
Age, years, median (IQR)	58 (47-63)	59 (51-69)	63
Age ≥65 years, No. (%)	6 (16)	11 (39)	0 (0)
Female sex, No. (%)	25 (68)	23 (82)	1 (100)
Preoperative chemotherapy, No. (%)	2 (5)	0 (0)	0 (0)
PCI score, median (IQR)	21 (13-30)	33 (18-36)	21
PCI score ≥20, No. (%)	20 (54)	20 (71)	1 (100)
EBL, mL, median (IQR)	250 (200-400)	535 (250-755)	NR
Intraoperative RBC transfusion, No. (%)	NR	NR	1 (100)
Length of surgery, hours, median (IQR)	6.6 (6.0-8.5)	7.0 (4.6-8.1)	5.7
HIPEC, No. (%)	29 (78)	28 (100)	1 (100)
HIPEC agent, No. (%)			
Mitomycin C	26 (90)	0 (0)	0 (0)
Doxorubicin + cisplatin	1 (3)	0 (0)	1 (100)
Mitomycin C + cisplatin	1 (3)	28 (100)	0 (0)
Cisplatin	1 (3)	0 (0)	0 (0)
CC score, No. (%)			
CC-0	21 (57)	12 (43)	1 (100)
CC-1	10 (27)	8 (29)	0 (0)
CC-2/3	6 (16)	8 (29)	0 (0)
Length of ICU stay, days, median (IQR)	NR	NR	2
Length of hospital stay, days, median (IQR)	7 (6-8)	16 (14-21)	12
30-day major morbidity, No. (%)	9 (24)	5 (18)	0 (0)
60-day mortality, No. (%)	2 (5)	2 (7)	0 (0)
Postoperative chemotherapy, No. (%)	1 (3)	0 (0)	1 (100)

Abbreviations: AN, appendiceal neoplasms; CC, completeness of cytoreduction; EBL, estimated blood loss; HIPEC, hyperthermic intraperitoneal chemotherapy; ICU, intensive care unit; NR, not reported; PCI, peritoneal cancer index; PSM, peritoneal surface malignancies.

Median follow-up was 11 (95% CI, 6 to 16) months at the Kyiv center. Median OS was not reached (NR; 95% CI, not available [NA]); median PFS was 27 (95% CI, 16 to 38) months. The 3-year OS rate was 79% and the 3-year PFS rate was 38%, while the 5-year survival rate was NA. At the Lviv center, median follow-up was 37 (95% CI, 21 to 53) months. Median OS was NR (95% CI, NA) and median PFS was 55 (95% CI, NA) months. Three-year and 5-year OS rates were 85% and 67%, respectively. Three-year and 5-year PFS rates were 65% and 49%, respectively.

### CRC

At Kyiv, Lviv, and Odesa centers, the median PCI scores were, respectively, 10 (IQR, 6-13), 10 (IQR, 6-17), and seven (IQR, 5-9; Table 4). The CC‐0/1 rates were 84%, 91%, and 86%, respectively. Postoperative 30-day major complications occurred in 16% (n = 3) at Kyiv, 18% (n = 2) at Lviv, and 8% (n = 12) at Odesa.

**TABLE 4:** Perioperative Characteristics of Patients With CRC

Variable	PSM Center		
	Kyiv (n = 19)	Lviv (n = 11)	Odesa (n = 156)
Age, years, median (IQR)	61 (49-68)	52 (41-62)	63 (55-68)
Age ≥65 years, No. (%)	7 (37)	1 (9)	63 (40)
Female sex, No. (%)	10 (53)	5 (46)	88 (56)
Preoperative chemotherapy, No. (%)	12 (63)	7 (64)	92 (59)
PCI score, median (IQR)	10 (6-13)	10 (6-17)	7 (5-9)
PCI score ≥20, No. (%)	3 (16)	1 (9)	6 (4)
EBL, mL, median (IQR)	200 (100-300)	NR	NR
Intraoperative RBC transfusion, No. (%)	NR	NR	66 (42)
Length of surgery, hours, median (IQR)	6.0 (5.0-6.5)	NR	3.8 (3.2-4.5)
HIPEC, No. (%)	8 (42)	11 (100)	15 (10)
HIPEC agent, No. (%)			
Mitomycin	7 (89)	11 (100)	3 (20)
Doxorubicin + cisplatin	0 (0)	0 (0)	12 (80)
Cisplatin	1 (13)	0 (0)	0 (0)
CC score, No. (%)			
CC-0	14 (74)	8 (73)	92 (59)
CC-1	2 (11)	2 (18)	42 (27)
CC-2/3	3 (16)	1 (9)	22 (14)
Length of ICU stay, days, median (IQR)	NR	1 (1-2)	1 (1-2)
Length of hospital stay, days, median (IQR)	8 (6-12)	NR	11 (9-15)
30-day major morbidity, No. (%)	3 (16)	2 (18)	12 (8)
60-day mortality, No. (%)	0 (0)	1 (9)	1 (1)
Postoperative chemotherapy, No. (%)	7 (37)	8 (73)	134 (86)

Abbreviations: CC, completeness of cytoreduction; CRC, colorectal cancer; EBL, estimated blood loss; HIPEC, hyperthermic intraperitoneal chemotherapy; ICU, intensive care unit; NR, not reported; PCI, peritoneal cancer index; PSM, peritoneal surface malignancies.

Only the Odesa cohort had ≥20 patients and underwent survival analysis. Median follow-up was 38 (95% CI, 34 to 42) months. Median OS was 35 (95% CI, 32 to 38) months and median PFS was 14 (95% CI, 13 to 15) months. Three-year and 5-year OS rates were 47% and 10%, respectively, while 3- and 5-year PFS rates were 5% and 3%, respectively.

### MPM

The median PCI scores were, respectively, 23 (IQR, 10-25), 26 (IQR, 16-32), and 10 (IQR, 7-15) at Kyiv, Lviv, and Odesa centers (Table 5). The CC‐0/1 rates were 67%, 80%, and 100%, respectively. Thirty-day major complications occurred in 13% (n = 2) of Kyiv, 0% (n = 0) of Lviv, and 17% (n = 1) of Odesa cases. We did not analyze survival because of small MPM cohorts at each center.

**TABLE 5:** Perioperative Characteristics of Patients With MPM

Variable	PSM Center		
	Kyiv (n = 15)	Lviv (n = 5)	Odesa (n = 6)
Age, years, median (IQR)	47 (33-58)	51 (40-55)	55 (43-68)
Age ≥65 years, No. (%)	0 (0)	0 (0)	2 (33)
Female sex, No. (%)	13 (87)	4 (80)	3 (50)
Preoperative chemotherapy, No. (%)	5 (33)	1 (20)	2 (33)
PCI score, median (IQR)	23 (10-25)	26 (16-32)	10 (7-15)
PCI score ≥20, No. (%)	8 (53)	3 (60)	1 (17)
EBL, mL, median (IQR)	200 (100-250)	NR	NR
Intraoperative RBC transfusion, No. (%)	NR	NR	0 (0)
Length of surgery, hours, median (IQR)	5.0 (4.0-6.0)	NR	4.6 (3.2-5.3)
HIPEC, No. (%)	13 (87)	5 (100)	6 (100)
HIPEC agent, No. (%)			
Doxorubicin + cisplatin	11 (85)	5 (100)	5 (83)
Carboplatin	0 (0)	0 (0)	1 (17)
Mitomycin + cisplatin	1 (8)	0 (0)	0 (0)
Cisplatin	1 (8)	0 (0)	0 (0)
CC score, No. (%)			
CC-0	6 (40)	3 (60)	2 (33)
CC-1	4 (27)	1 (20)	4 (67)
CC-2/3	5 (33)	1 (20)	0 (0)
Length of ICU stay, days, median (IQR)	NR	1 (1-1)	1 (1-2)
Length of hospital stay, days, median (IQR)	7 (7-8)	NR	9 (7-14)
30-day major complications, No. (%)	2 (13)	0 (0)	1 (17)
60-day mortality, No. (%)	0 (0)	0 (0)	0 (0)
Postoperative chemotherapy, No. (%)	0 (0)	2 (40)	4 (67)

Abbreviations: CC, completeness of cytoreduction; EBL, estimated blood loss; HIPEC, hyperthermic intraperitoneal chemotherapy; ICU, intensive care unit; MPM, malignant peritoneal mesothelioma; NR, not reported; PCI, peritoneal cancer index; PSM, peritoneal surface malignancies.

### EOC

#### Kyiv Center

The CC‐0/1 rates were 79% and 80% in primary and recurrent EOC, respectively (Table 6). The 30-day major morbidity rates were 23% (n = 21) and 20% (n = 2), respectively. Survival analysis was performed only for primary EOC because of small recurrent subgroup. Median follow-up was 4 (95% CI, 3 to 5) months, median OS—NR (95% CI, NA), and median PFS—11 (95% CI, 8 to 14) months. One- and 3-year OS rates were 86% and 80%, respectively, while 1- and 3-year PFS rates were 47% and 0%, respectively. Five-year survival rate was NA.

**TABLE 6:** Perioperative Characteristics of Patients With EOC

Variable	PSM Center					
	Kyiv		Lviv		Odesa	
	Primary (n = 91)	Recurrent (n = 10)	Primary (n = 40)	Recurrent (n = 87)	Primary (n = 89)	Recurrent (n = 1)
Age, years, median (IQR)	57 (49-63)	56 (50-63)	58 (51-66)	54 (50-58)	57 (48-65)	43
Age ≥65 years, No. (%)	18 (20)	1 (10)	12 (30)	8 (9)	24 (27)	0 (0)
Preoperative chemotherapy, No. (%)	28 (31)	1 (10)	38 (95)	17 (20)	22 (25)	1 (100)
PCI score, median (IQR)	18 (10-25)	13 (8-20)	8 (5-14)	11 (5-20)	10 (7-14)	24
PCI score ≥20, No. (%)	34 (37)	3 (30)	1 (3)	23 (26)	8 (9)	1 (100)
EBL, mL, median (IQR)	300 (200-400)	300 (200-300)	NR	NR	NR	NR
Intraoperative RBC transfusion, No. (%)	NR	NR	NR	NR	43 (48)	0 (0)
Length of surgery, hours, median (IQR)	5.5 (4.2-7.0)	4.7 (4.1-6.4)	NR	NR	2.8 (2.3-3.8)	2.3
HIPEC, No. (%)	40 (44)	3 (30)	40 (100)	87 (100)	17 (19)	0 (0)
HIPEC agent, No. (%)						
Doxorubicin + cisplatin	1 (3)	0 (0)	0 (0)	32 (37)	17 (100.0)	NA
Cisplatin	39 (98)	3 (100)	40 (100)	55 (63)	0 (0)
CC score, No. (%)						
CC-0	49 (54)	7 (70)	33 (83)	58 (67)	47 (53)	0 (0)
CC-1	23 (25)	1 (10)	7 (18)	14 (16)	24 (27)	0 (0)
CC-2/3	19 (21)	2 (20)	0 (0)	15 (17)	18 (20)	1 (100)
Length of ICU stay, days, median (IQR)	NR	NR	1 (1-1)	1 (1-2)	1 (1-1)	1
Length of hospital stay, days, median (IQR)	8 (7-10)	7 (7-10)	NR	NR	8 (7-11)	16
30-day major complications, No. (%)	21 (23)	2 (20)	2 (5)	23 (26)	5 (6)	1 (100)
60-day mortality, No. (%)	1 (1)	0 (0)	0 (0)	5 (6)	2 (2)	0 (0)
Postoperative chemotherapy, No. (%)	34 (37)	1 (10)	39 (98)	54 (62)	81 (91)	1 (100)

Abbreviations: CC, completeness of cytoreduction; EBL, estimated blood loss; EOC, epithelial ovarian, fallopian tube, and primary peritoneal cancer; HIPEC, hyperthermic intraperitoneal chemotherapy; ICU, intensive care unit; NA, not applicable; NR, not reported; PCI, peritoneal cancer index; PSM, peritoneal surface malignancies.

#### Lviv Center

The CC‐0/1 rates were 100% (n = 40) and 83% (n = 72) in primary and recurrent EOC, respectively (Table 6). Thirty-day major complications occurred in 5% (n = 2) and 26% (n = 23), respectively. Median follow-up was 36 (95% CI, 29 to 43) months. Median OS in the primary and recurrent subgroups was 71 (95% CI, 32 to 110) and 58 (95% CI, 43 to 73) months, respectively. One-, 3-, and 5-year OS rates in primary EOC were 97%, 72%, and 50%, respectively. One-, 3-, and 5-year OS rates in recurrent EOC were 87%, 61%, and 45%, respectively. PFS data were not reported for EOC in the Lviv data set.

#### Odesa Center

We included 89 primary and one recurrent EOC patient (her characteristics are in Table 6). The CC-0/1 rate was 80% (n = 71). Thirty-day major complications occurred in 6% (n = 5). Survival analysis was performed only for primary EOC. Median follow-up was 57 (95% CI, 49 to 65) months, median OS—67 (95% CI, 61 to 73) months, and median PFS—31 (95% CI, 28 to 34) months. One-, 3-, and 5-year OS rates were 92%, 79%, and 69%, respectively, while 1-, 3-, and 5-year PFS rates were 92%, 33%, and 0%, respectively.

## DISCUSSION

To our knowledge, this study is the first to describe the current state of the PSM care system in the lower-middle–income setting of Ukraine.^15^ Our assessment of three active CRS/HIPEC programs demonstrated favorable short- and long-term outcomes aligning with global standards for PSM expert centers.^16^ These compelling findings prompted us to examine essential elements of each program highlighting existing barriers and uncovering the strategies to establish effective CRS/HIPEC programs in countries facing similar challenges (Table 7).

**TABLE 7:** Strategies to Overcome Common Barriers to Establishing a PSM Center in Lower-Middle–Income Settings

Category	Barrier	Possible Strategy
Resources	Insufficient PSM program funding	Collaborative development of national guidelines on PSM management by medical professionals and health care officials to secure funding
		Fundraising events and private sponsorship
		Out-of-pocket payments
	Unavailability of 24/7 CT scan	On-call radiologist services for emergency situations
		Remote access to images
		Specialized imaging training for PSM surgeons and residents
	Absence of interventional radiology	Training of PSM surgeons and radiologists in US-/CT-guided drainage procedures
	Shortage of nutrition, OT, and PT specialists	Telemedicine
	No dedicated research department	Allocation of dedicated research time for surgeons and residents
		Starting PSM program at academic centers
Referral	Limited PSM knowledge among physicians	Development of national guidelines on PSM management
		Incorporation of PSM topics into medical school and residency curricula
		Educational materials for oncological and nononcological specialists by professional societies
		Engaging PSM specialists in nononcological (GI, colorectal, and gynecologic) events
		Development of in-person and online platforms for multidisciplinary communication and education
		Creation of regional networks connecting referral bases to the nearest PSM center
		Development of the national CRS/HIPEC registry with transparent outcomes
		Presentation and publication of the outcomes
	Public's lack of awareness of PSM and CRS/HIPEC	Creation of patient-advocating NGO and their interaction with PSM specialists
		Development of an online national registry of PSM centers and surgeons
		Implementation of community awareness programs
		Online publication of easy-to-read educational content
	Lack of access to PSM centers	NGO financial assistance for patient transportation
		Development of new PSM programs
Training	Deficiency in PSM and CRS/HIPEC training	International observership for surgeons, medical oncologists, pathologists, and radiologists
		Partnership with international PSM centers
		Intensive 2-year ESSO program for aspiring PSM surgeons
		Collaboration with established PSM centers nationally
		Peer-learning within one institution
Follow-up	Inadequate funding for post-treatment follow-up	Collaborative development of national guidelines on PSM management by medical professionals and health care officials to secure funding
		Coding of AN cases as CRC for billing purposes
		Practical adjustments to follow-up schedules and diagnostic tools
		Involvement in RCTs subsidizing surveillance costs
		Out-of-pocket payments
	Inadequate access to PSM centers	NGO financial assistance for patient transportation
		Telemedicine
	Limited PSM expertise among regional oncologists	Establishment of national guidelines on PSM management
		Detailed surveillance schedule within the patient discharge note with the PSM surgeon contacts
		Incorporation of PSM topics into medical school and residency curricula
		Educational resources from professional societies for oncological and nononcological specialists

Abbreviations: AN, appendiceal neoplasms; CRC, colorectal cancer; CRS/HIPEC, cytoreductive surgery/hyperthermic intraperitoneal chemotherapy; CT, computed tomography; ESSO, European Society of Surgical Oncology; NGO, nongovernmental organization; OT, occupational therapy; PSM, peritoneal surface malignancies; PT, physical therapy; RCT, randomized controlled trial; US, ultrasound.

**FIG 1:**
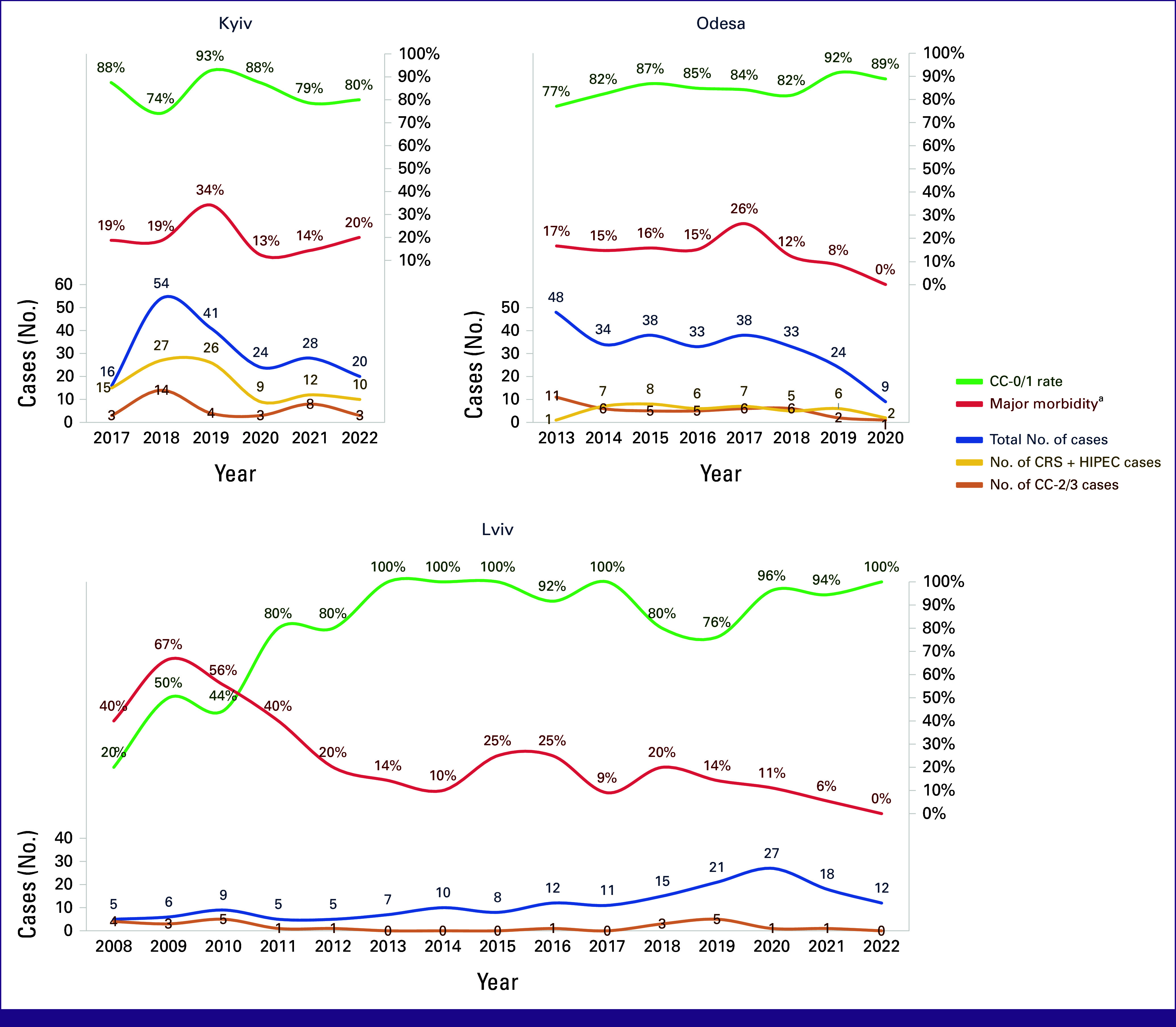
Changes in caseload, CC-0/1 rate, and major morbidity^a^ rate at PSM centers. All presented cases at the Lviv center are CRS + HIPEC. ^a^Grade III-IV complications by the Clavien-Dindo classification. CC, completeness of cytoreduction; CRS, cytoreductive surgery; HIPEC, hyperthermic intraperitoneal chemotherapy; PSM, peritoneal surface malignancy.

Efficient PSM programs involve multiple specialists and services, driving the costs of CRS/HIPEC and perioperative care up to $80,000 (US dollars) per patient in advanced economies.^2,3,8,16,23-26^ This would be a significant financial burden on the LMIC health care system, especially when it is government-funded like in Ukraine.^27^ Although public health care has its merits, it can be reluctant to implementation of novel modalities.^28,29^ As a result, new expenses not accounted for by the funding partially fall on patients as observed at the Kyiv and Lviv centers (Table 1). This issue also explains limited HIPECs in Odesa (Fig 1): government coverage extends only to billable CRS components, not HIPEC-related costs. To address these challenges, we recommend codeveloping national guidelines with health care officials to facilitate the inclusion of new therapies within government budgets.^30^

Although certain PSM program's components such as surgical ICU and blood bank are indispensable for perioperative management, others offer some flexibility.^16,31^ For example, instead of having radiologists on site 24/7, emergency calls or remote access to images can suffice. Additionally, with training, PSM surgeons and residents can interpret ultrasound (US) and CT scans.^32-34^ For centers without specialized interventional radiology, short courses can enable radiologists and surgeons to perform predominant post-CRS/HIPEC procedures—US or CT-guided drainage of intrapleural and intra-abdominal fluid collections.^35-38^ This flexibility in service delivery is underscored by the similar morbidity rates across our centers, irrespective of their resources (Tables 3-6). Finally, the delay in CRS/HIPEC data recording, as seen at the Odesa center (Fig 1) and attributed to staffing limitations, can be addressed by allocating secured research time to surgeons and residents, thereby also encouraging them to continue research further in their careers.^39^

Successful PSM management relies on timely referrals to specialized CRS/HIPEC centers as they allow for superior outcomes.^10,40^ Our analysis revealed volatile patient dynamics across Ukrainian centers and highlighted several referral challenges (Fig 1). Unfamiliarity with CRS/HIPEC is a major barrier to its adoption in both low- and high-income countries.^12,41-44^ Ukrainian and international professional societies offer valuable educational resources to bridge this knowledge gap, yet nononcology specialists also frequently encounter patients with PSM and require familiarization.^45-48^ Since the main obstacle to CRS/HIPEC adoption and referral is lack of interaction with cytoreductive surgeons, we suggest inviting PSM specialists to share their expertise at various, not only oncologic, conferences and platforms.^44,49-51^

Our data indicated a skew toward referrals of more prevalent and familiar CRC and EOC, while AN and MPM caseloads stayed low.^52,53^ In Ukraine, this was addressed by the development of national guidelines, a lack of which is another known barrier to patient referral.^30,41,43^ Integrating PSM topics into oncology residency or medical school curricula can further cement this knowledge. Addressing misconceptions about CRS/HIPEC safety and efficacy is equally important.^43,50^ Besides networking with PSM surgeons, referring physicians might alleviate their concerns with a national CRS/HIPEC registry publishing transparent outcomes of PSM centers.^41,44,51^

As patients and their families often independently seek new treatments, their awareness is also crucial.^1,42,44,53^ Here, patient-advocating nongovernmental organizations (NGOs) play a role in facilitating physician-patient interaction, providing educational content, psychological support, and even financial assistance for transportation, thereby addressing the PSM center inaccessibility barrier.^41,43,54-56^ Launching new CRS/HIPEC programs is another recommended yet resource-intensive way to deal with low access: initial significant caseloads at the Odesa and Kyiv centers showcase how unmet the demand was in these regions serviced before solely by the center in Lviv (Fig 1).^41^

Any PSM program foundation rests on the surgeons training, which these programs typically start with.^3,40^ Given the substantial learning curve of 90-180 surgeries for CRS and the absence of dedicated PSM fellowship programs, alternative strategies are vital.^57-61^ The path to CRS/HIPEC should start with a solid background in general surgery and surgical oncology, while training in gynecology or gynecologic oncology can also be advantageous as EOC is a common PSM.^2,62^ Then, mastering the technical and managerial complexities of CRS/HIPEC and perioperative care often involves seeking guidance from reputed PSM centers.^61^ International observerships provide good initial exposure; however, they are generally brief and costly, and gained experience may not be entirely applicable in LMICs.^63,64^ A more comprehensive approach is the 2-year program from the European Society of Surgical Oncology encompassing hands-on experience, tumor boards, follow-up, and research, along with fostering long-term multi-institutional partnership.^65-67^

Long-term experience exchange between centers within one country has been shown to expedite the CRS/HIPEC learning curve.^68^ Our observations align with this, noting the swift compliance with Chicago Consensus quality standards of the newer Odesa and Kyiv centers compared with the pioneering Lviv center (Fig 1).^16^ Cooperation and peer learning in the operating room is also important, allowing newcomers to attain proficiency similar to experienced surgeons faster than learning solo.^2^ Such strategies ensure adherence to global standards in CC-0/1, morbidity, mortality, and length of hospital stay (Tables 3-6), and pave the way for developing national training programs that take into account both global evidence and local context.^16,69-75^

Regular follow-up is paramount because of the significant recurrence rate, even after successful treatment.^76-78^ Intraperitoneal relapse is predominant, and dedicated surveillance can improve survival through iterative surgery feasibility.^79-82^ Although organizing long-lasting follow-up is challenging, in LMICs, codeveloped national guidelines mentioned before can both equip local oncologists with surveillance protocols and support budget allocations. While these guidelines are in progress, a temporary solution can be found in following and billing rare AN cases as CRC.^83,84^

Distance and financial constraints limit patients' ability to revisit their PSM specialists for follow-ups. Although some patients self-fund their trips, rely on NGOs, or resort to telemedicine, a significant proportion is followed by their regional oncologists.^85^ Therefore, we suggest providing a detailed surveillance schedule within the patient discharge note with the surgeon's contacts. This collaborative approach elevates care standards, stimulates cooperation between involved specialists, and raises PSM awareness, potentially increasing future referrals.^49-51^ A dedicated PSM research department offers another way for diligent patient monitoring as it mandates regular follow-up and allows participation in international studies with thorough participant surveillance.^2,86^ However, in LMICs, conducting research solely by enthusiastic physicians can be a viable approach as evidenced by the median follow-up and survival rates at Lviv and Odesa centers consistent with world data.

Most PSMs relapse within 2-3 years of treatment, which helps to tailor the surveillance tools and schedule.^76-78,87^ Abdominal and pelvic CT has the highest accuracy for PSM; however, resource constraints can limit its availability.^88,89^ In such cases, US becomes an option, when performed by experienced operators, especially accurate for diaphragmatic, splenic, omental, and pelvic lesions.^90-93^ If CT requires long waiting, an alternating schedule with US could be implemented. Intrathoracic recurrence is rarer than intraperitoneal yet still possible; therefore, chest imaging is required, and when CT is unavailable, chest X-ray could be considered.^35,94-96^ In addition, a less frequent follow-up schedule every 6-12 months for AN and CRC has been shown to offer significant economic relief and still be as effective as surveillance every 2-4 months without compromising patient outcomes.^97^

This study has several limitations because of its retrospective and descriptive design. Our objective was an analytical review of the PSM centers' strengths and challenges rather than a direct comparison. The used data sets did not distinguish histologic subtypes of AN and they were analyzed together. The lack of follow-up at the Kyiv center did not allow us to make solid conclusions on the long-term outcomes. In turn, the Lviv center had no PFS data on included patients with EOC; its data set also had no records of EOC cases treated with CRS only.

In conclusion, this study offers a comprehensive examination of PSM management in Ukraine. Despite facing systemic challenges, all three CRS/HIPEC centers demonstrate promising outcomes aligned with global standards. Multidisciplinary collaboration, adaptability, and resourcefulness have been instrumental in achieving these results. Our work highlights the importance of enhancing PSM and CRS awareness among physicians and the public, expanding referral networks, and fostering research for continuous improvement. We believe the provided insights and pathways will help physicians surmount barriers common in LMICs and enable more patients to receive top-tier care.
